# Preoperative staging of uterine cancer: can transvaginal ultrasonography play a role?

**DOI:** 10.3389/fonc.2023.1089105

**Published:** 2023-06-19

**Authors:** Mariana Rei, Cristina Costa-Santos, João Bernardes, Antónia Costa

**Affiliations:** ^1^ Department of Gynecology, Instituto Português de Oncologia Francisco Gentil, Porto, Portugal; ^2^ Department of Obstetrics, Gynecology and Pediatrics, Medical School, University of Porto, Porto, Portugal; ^3^ Department of Health Informatics and Decision Sciences, Medical School, University of Porto, Porto, Portugal; ^4^ Centre for Research in Health Information Systems and Technologies, CINTESIS, University of Porto, Porto, Portugal; ^5^ Department of Obstetrics and Gynecology, Centro Hospitalar Universitário de São João, Porto, Portugal

**Keywords:** uterine cancer, endometrial neoplasia, pelvic ultrasound, myometrial invasion, cervical invasion, staging

## Abstract

**Introduction:**

Preoperative staging of uterine cancer has recently been implied as an important contribution to an accurate selection of low-risk cases, ultimately avoiding unnecessary lymph node debulking. The aim of this study was to evaluate the validity of transvaginal ultrasonography (TVS) in preoperative staging of uterine cancer in comparison to pelvic magnetic resonance imaging (MRI) and permanent section.

**Methods:**

We conducted a prospective longitudinal multicenter trial between 2017 and 2018. Inclusion criteria comprised cases of endometrial neoplasia histologically confirmed or strong imaging suspicion, candidates for elective surgery as primary treatment. Proportions of Agreement (PA), kappa statistic (K), sensitivity, specificity and accuracy were calculated with 95% confidence intervals (95%CI).

**Results:**

Eighty-two patients were eligible for the study, presenting a mean age of 68 years (standard deviation 11). In what concerns the TVS evaluation of myometrial invasion, the subjective and objective methods of Gordon and Karlsson presented a sensitivity of 79%, 79% and 67% [95%CI 63-91; 63-91; 50-81], a specificity of 65%, 58% and 79% [95%CI 49-79; 42-73; 64-89] and an overall accuracy of 72%, 68% and 73% [95%CI 61-81; 57-78; 63-82]. MRI presented respectively a sensitivity, specificity and overall accuracy of 92%, 70% and 82% [95%CI 77-98; 52-85; 71-90]. Regarding cervical involvement, the sensitivity was respectively 31%, 50% and 67% [95%CI 9-61; 21-79; 35-90] for the subjective method, objective TVS and MRI, and the specificity was 98%, 90% and 100% [95%CI 92-100; 77-97; 94;100]. Agreement between TVS and MRI was superior in the assessment of cervical invasion, with PA ranging from 0.82 to 0.93 and K from 0.45 to 0.58, in comparison to myometrial invasion with PA ranging from 0.68 to 0.73 and K from 0.31 to 0.50. Considering the assessment of cervical involvement, as MRI showed a specificity of 100% it is not possible to increase the specificity. However, it was possible to increase the sensitivity, considering the combination of TVS with objective approach and MRI.

**Conclusion:**

TVS may have a promising role as a tool for preoperative staging of endometrial carcinoma, presenting a performance that approximates to MRI, with a higher agreement in the assessment of cervical invasion.

## 1 Introduction

Endometrial carcinoma is the sixth most common malignancy diagnosed in women and the most common gynecological cancer in high income countries, with 417.000 new diagnosis globally in 2020 (1) and a 5-year relative survival of 76% according to the EUROCARE-5 study (2).

Classically, endometrial cancer was a surgically staged disease, and therefore routine preoperative work-up to assess myometrial invasion or cervical involvement was often considered unnecessary. With the advent of the sentinel node technique, lymph node biopsy is an acceptable alternative to systematic lymphadenectomy in stage I/II, therefore avoiding lymphadenectomy-related morbidity (3, 4).

Major prognostic features for endometrial carcinoma include grading, histotype, nodal metastasis and deep myometrial infiltration (5). Particularly, myometrial infiltration and cervical involvement, which can be assessed by ultrasound, are key preoperative parameters, that might alter staging and surgical approach (6, 7).

As the frozen section is now discouraged for myometrial invasion assessment due to poor reproducibility, and sentinel node biopsy is increasingly used for lymph node staging in stage I/II endometrial cancer (4), the use of the most suitable preoperative work-up is a fundamental feature for surgical planning and correct inform consent.

Our study aimed to evaluate the validity of transvaginal ultrasonography (TVS) in preoperative staging of endometrial cancer, especially regarding myometrial and cervical stromal invasion, in comparison to the gold-standard of tumor staging which is final histology. We also aimed to determine the agreement between TVS and pelvic magnetic resonance imaging (MRI), the established standard of care in pre-surgical imaging staging.

## 2 Materials and methods

This is a prospective longitudinal multicenter trial conducted between October 2017 to December 2018 in three portuguese hospitals: Centro Hospitalar de São João, in Oporto, Instituto Português de Oncologia in Coimbra and Hospital Beatriz Ângelo, in Loures. The patient enrollment comprised women referred to gynecological oncology consultation in one of these three centers due to uterine cancer suspicion. Women with histologically confirmed endometrial malignancy (by dilation and curettage, hysteroscopy or endometrial biopsy) or strong imaging suspicion and planned surgery as primary treatment were eligible to participate in the study. Suspicion of uterine malignancy without histological confirmation, contraindication to undergo pelvic MRI, technical issues not allowing transvaginal or transrectal ultrasonography, contraindication to surgery as primary treatment, urgent or emergency life-saving surgery not allowing pre-surgical diagnosis and staging were considered exclusion criteria. The protocol and consent forms were approved by the institutional review board of each center, and all participants or their legally authorized representative provided written informed consent before enrollment.

### 2.1 Recruitment protocol

Recruitment for the trial and data collection was delivered as one-to-one consultation by the principal investigator.

The diagnostic features collected, while performing TVS, followed a designated protocol based on previous studies (6, 8, 9) which included: uterine and tumor dimensions and volume; endometrial thickness; intracavitary fluid; tumor vascularization; regularity of the endometrial contour and junctional zone; myometrial invasion using the subjective approach (based on evaluation of disrupted endometrial/myometrial border and a subjective correlation of the width of myometrium along with the depth of tumour invasion) and the objective assessment by Gordon ((the distance between the maximum tumour depth and the total myometrial thickness) and Karlsson methods (the maximum anteroposterior thickness of the endometrial lesion measured in the sagittal plane divided by the anteroposterior uterine diameter) (10, 11); cervical involvement (subjective assessment and objective method using a cut-off less than 20mm between the outer cervical orifice to the lower margin of the tumor) (6, 12); invasion of adjacent organs; ascites; other gynecological findings prone for altering the staging. MRI criteria were defined by the radiologist expert based on international consensus in endometrial cancer imaging staging (13).

TVS was either performed by a resident fellow (5 years of training) or a specialist (more than 5 years of expertise in gynecologic imaging). The equipment to employ was a GE Voluson E8 (GE Medical systems Austria) US system, equipped with RICS5-9 transducer, or equivalent device. Pelvic MRI was interpreted by a radiology specialist with more than 5 years of expertise in gynecologic imaging (one per center). All imaging exams were blinded for the remaining clinical and imaging data and systematically followed a predefined protocol, filled out by the investigator after each examination.

Comprehensive surgical staging comprise abdominopelvic washings, total hysterectomy, bilateral salpingo-oophorectomy and pelvic and para-aortic lymph node dissection according to intra-operative frozen section staging or histologic criteria in preoperative biopsy (14). Histologic assessment following surgery was considered the gold standard. Post-operative staging was performed by a dedicated pathologist with expertise in gynecologic oncology, based on the most recent FIGO 2009 staging guidelines (15).

### 2.2 Statistical analysis

Sensitivity, specificity and overall accuracy were calculated, with ninety-five percent confidence intervals (95% CI) for each staging method (TVS or MRI), in comparison to the final histopathological exam, that was considered the gold standard. These calculations evaluated myometrium and cervical invasion once these are key features that might impact staging and different surgical approaches.

Agreement was be assessed by overall and specific Proportions of Agreement (PA) from each category and reliability with the kappa statistic (K). If the lower limit of the 95% CI for the PA is under 0.50, agreement will be considered poor (16). PA for a specific category estimates the conditional probability, given that one of the methods makes a rating in that category, the other method will also do so. A K value lower than 20 was considered poor reliability; it was categorized by fair between 0.21 and 0.40, moderate between 0.41 and 0.60, substantial between 0.61 and 0.80 and excellent if higher than 0.80 (17).

## 3 Results

Eighty-two patients were eligible for the study, presenting a mean age of 68 years (standard deviation [sd] =11): 88% postmenopausal (72/82), 66% with arterial hypertension (54/81), 57% obese (47/82), 26% diabetic (21/82) and 11% with high risk endometrial cancer mutations (9/82). The majority of endometrial cancers included in our study were of endometrioid histological type (79%, 65/82) of which 78% (51/65) were well to moderately differentiated (grade 1 or 2). Most malignancies were diagnosed at FIGO stage 1 or 2 (66/82, 80%) (**Table 1**).

**Table 1:** Population characteristics. sd: standard deviation.

	n	mean (sd) or n (%)
Age *mean (sd)*	82	68 (11)
Age of menopause mean (sd)	72	50 (6)
Body mass index (kg/m^2^) mean (sd)	79	32 (7)
Type 2 Diabetes mellitus n (%)	82	21 (26)
Arterial hypertension n (%)	81	54 (66)
BRCA or Lynch mutation n (%)	82	9 (11)
Histology n (%)	82	
Endometroid		65 (79.3)
Serous		9 (11.0)
Clear cells		1 (1.2)
Mixed		2 (2.4)
Undifferentiated		2 (2.4)
Carcinossarcoma		3 (3.7)
FIGO Stage n (%)	82	
IA		35 (43)
IB		23 (28)
II		8 (10)
III		16 (19)

Regarding myometrial invasion, we found a sensitivity of 79%, 79% and 67% and a specificity of 65%, 58% and 79% for the subjective, Gordon and Karlsson approaches respectively. An overall accuracy of 72%, 68% and 73% was found respectively for the subjective, Gordon and Karlsson methods. Contrast-enhanced MRI provided a sensitivity, specificity and overall accuracy of 92%, 70% and 82% for myometrial invasion (**Table 2**).

**Table 2:** Sensitivity, specificity and accuracy of TVS and MRI in the evaluation of myometrial invasion.

Myometrial invasion	N	Sensitivity [95%CI]	Specificity [95%CI]	Accuracy [95%CI]
TVS Subjective method	82	0.79 [0.63; 0.91]	0.65 [0.49;0.79]	0.72 [0.61; 0.81]
TVS Gordon method	82	0.79 [0.63; 0.91]	0.58 [0.42; 0.73]	0.68 [0.57; 0.78]
TVS Karlsson method	82	0.67 [0.50; 0.81]	0.79 [0.64; 0.89]	0.73 [0.63; 0.82]
MRI	70	0.92 [0.77; 0.98]	0.70 [0.52; 0.85]	0.82 [0.71; 0.90]

Considering the evaluation of cervical stromal invasion, we found a sensitivity and specificity of 31% and 98% for the ultrasound subjective approach and 50% and 90% for the objective method; overall accuracy reached respectively 88% and 81% for both methods. Contrast-enhanced MRI provided a sensitivity, specificity and overall accuracy of 67%, 100% and 94% for cervical involvement (**Table 3**).

**Table 3:** Sensitivity, specificity and accuracy of TVS and MRI in the evaluation of cervical invasion.

Cervical invasion	N	Sensitivity [95%CI]	Specificity [95%CI]	Accuracy [95%CI]
TVS Subjective method	82	0.31 [0.09; 0.61]	0.98 [0.92; 1.00]	0.88 [0.79; 0.94]
TVS Objective method	53	0.50 [0.21; 0.79]	0.90 [0.77; 0.97]	0.81 [0.68; 0.90]
MRI	70	0.67 [0.35;0.90]	1.00 [0.94; 1.00]	0.94 [0.86; 0.98]

The reliability between MRI and TVS for the three ultrasound approaches was fair to moderate (K= 0.31 to 0.50) in the evaluation of myometrial deep invasion and moderate (K= 0.45 to 0.58) regarding cervical involvement. Agreement in the assessment of cervical invasion was better for “no” category than for “yes” (**Table 4**).

**Table 4:** Reliability and agreement between TVS and MRI regarding myometrial deep invasion and cervical involvement.

Myometrial invasion	kappa [95%CI]	PA [95%CI]
TVS Karlsson method vs MRI	0.50 [0.30; 0.70]	0.74 [0.63; 0.84]
Superficial		0.57 [0.41; 0.72]
Deep		0.61 [0.45; 0.74]
TVS Subjective method vs MRI	0.43 [0.21; 0.65]	0.73 [0.61; 0.82]
Superficial		0.49 [0.32; 0.65]
Deep		0.63 [0.49; 0,76]
TVS Gordon method vs MRI	0.31 [0,07; 0.54]	0.68 [0.55; 0.78]
Superficial		0.39 [0.24; 0.56]
Deep		0.59 [0.45; 0.72]
Cervical invasion		
TVS Subjective method vs MRI	0.58 [0.22; 0.93]	0.93 [0.83; 0.97]
No		0.92 [0.82; 0.97]
Yes		0.44 [0.15; 0.77]
TVS Objective method vs MRI	0.45 [0.10; 0.79]	0.82 [0.67; 0.91]
No		0.80 [0.64; 0.90]
Yes		0.38 [0.15; 0.68]
TVS Subjective vs Objective method	0.62 [0.30; 0.94]	0.90 [0.78; 0.96]
No		0.89 [0.76; 0.96]
Yes		0.50 [0.20; 0.96]

While combining techniques, we found a sensitivity of 75%, 72% and 67% and a specificity of 82%, 82% and 88% for the subjective, Gordon and Karlsson approaches respectively for the evaluation of myometrial invasion, when these approaches were combined with MRI for a positive deep myometrial invasion. If at least one approach classifies as positive deep myometrial invasion, we found a sensitivity of 97%, 100% and 94% and a specificity of 50%, 44% and 65% for the subjective, Gordon and Karlsson approaches respectively when combined with MRI (**Table 5**).

**Table 5:** Sensitivity, specificity and accuracy of combination of TVS and MRI (“yes” if both approaches classify “yes” and “yes” if at least one approach classifies “yes”) in the evaluation of myometrial invasion.

Myometrial invasion	N	Sensitivity [95%CI]	Specificity [95%CI]	Accuracy [95%CI]
“yes” if both approaches classify “yes”				
TVS Subjective method and MRI	70	0.75 [0.58; 0.88]	0.82 [0.65; 0.93]	0.78 [0.67; 0.87]
TVS Gordon method and MRI	70	0.72 [0.55; 0.86]	0.82 [0.65; 0.93]	0.77 [0.66; 0.86]
TVS Karlsson method and MRI	70	0.67 [0.49; 0.81]	0.88 [0.72; 0.97]	0.77 [0.66; 0.86]
“yes” if at least one approach classifies “yes”				
TVS Subjective method and MRI	70	0.97 [0.85; 0.99]	0.50 [0.32;0.67]	0.74 [0.62; 0.84]
TVS Gordon method and MRI	70	1.00 [0.90; 1.00]	0.44 [0.27; 0.62]	0.73 [0.61; 0.83]
TVS Karlsson method and MRI	70	0.94 [0.81; 0.99]	0.65 [0.46; 0.80]	0.80 [0.60; 0.89]

## 4 Discussion

Imaging has been advocated in the preoperative management of patients with uterine corpus cancer (6). The recently reviewed European guidelines for the management of patients with endometrial carcinoma include expert transvaginal or transrectal ultrasound or pelvic MRI in the pre-operative mandatory work-up (4).

Regarding myometrial invasion, in our study MRI presented the highest sensitivity for myometrial invasion assessment (92%). We found an overlapping sensitivity for the subjective and Gordon approach (79%); Karlsson approach displayed the lowest sensitivity (67%) but the highest specificity (79%). If both TVS and MRI preoperative exams classify as positive to deep myometrial invasion, we report a combined sensitivity of 75%, 72% and 67% and a specificity of 82%, 82% and 88% for the subjective, Gordon and Karlsson approaches respectively. If at least one approach classifies as positive deep invasion, we found a higher sensitivity (97%, 100% and 94%) and lower specificity (50%, 44% and 65%) for the subjective, Gordon and Karlsson approaches respectively when combined with MRI. Of notice, MRI displayed a higher overall accuracy compared to the ultrasonographic methods (82% versus 68 – 73%).

A systematic review and meta-analysis of 24 studies reviewing the diagnostic accuracy of TVS in the preoperative detection of deep myometrial infiltration (18) revealed an overall pooled sensitivity and specificity of respectively 82% and 81%, with no statistical differences between subjective and objective methods.

Alcazar et al. conducted a trial comparing the diagnostic performance of six different approaches for assessing myometrial infiltration using transvaginal or transrectal ultrasound in women with grade 1 or 2 endometrioid carcinoma, including the impression of examiner, Karlsson’s criteria, endometrial thickness, tumor/uterine 3D volume ratio, tumor distance to myometrial serosa and van Holsbeke’s subjective model. The impression of examiner and subjective model seemed to be the best approaches for assessing myometrial infiltration (sensitivity of 79.5% and 80.5% and specificity of 89.6% and 90.3% respectively), both with significantly better sensitivity than Karlsson’s criteria (sensitivity 31.8%) (19). Frühauf et al. highlighted similar results, reporting a sentitivity, specificity and overall accuracy of respectively 79.3%, 73.2 and 75.7% for subjective assessment, 69.6%, 65.9% and 67.3% for Gordon’s ration and 56.3%, 76.4% and 68.1% for Karlsson’s approach (20). Our results reinforce these studies: the subjective approach yielded a high diagnostic overall accuracy (72%) and Karlsson’s criteria performed poorer than subjective evaluation in terms of sensitivity (67% vs 79%).

In a recent systematic review and meta-analysis, Alcazar et al. compared MRI and TVS for detecting myometrial infiltration in endometrial carcinoma, reporting a pooled estimated sensitivity and specificity of 75% and 82% for TVS, and 83% and 82% for MRI, respectively (21). Our study found comparable accuracies for both preoperative methods.

Agreement regarding deep myometrial invasion between preoperative transvaginal ultrasound and intraoperative macroscopic examination in low-risk endometrioid carcinoma was evaluated in an observational study comprising 152 women; although the agreement between the two approaches was only moderate, both methods had similar accuracy when compared with frozen section histology, reinforcing the use of preoperative ultrasound by expert (22).

Concerning other factors affecting the preoperative staging, Fischerova et al. did not confirm the expected correlation between ultrasound failure and obesity, uterus position or the quality of ultrasound imaging (23). A recent retrospective analysis indicates FIGO stage IB as the main significant confounding factor, with worse diagnostic accuracy in patients with concomitant benign uterine pathologies as diffuse fibromatosis and adenomyosis (24). In regard to the use of 3D transvaginal ultrasound, it did not show improved diagnostic accuracy of myometrial infiltration in comparison to 2D ultrasound in a large multicentre study (25). However, a recent systematic review reported that 3D-TVUS is at least comparable, in fact superior in one study, to MRI and mostly equivalent to 2D-TVUS, thus underlining a promising role (26).

A total of four examiners participated in the study, but due to a low number of exams performed by each physician it was not possible to understand slight differences in the overall diagnostic accuracy rate per physician and potential differences between experts and residents. Concerns regarding the reproducibility of the different measure techniques in real-time ultrasonography examinations remain. Ericsson et al. demonstrated a higher degree of agreement with histopathology and greater interobserver reproducibility in the assessment of cervical stromal invasion among ultrasonography experts, but not of deep myometrial invasion (27).

Regarding cervical stromal invasion, our data shows that MRI provided the highest specificity for cervical involvement (67%). The ultrasound subjective approach displayed a low sensitivity, but a high specificity (98%), slightly overcome by MRI; the objective methodology reached similar results for specificity, but with higher sensitivity compared to the subjective approach. Overall accuracy was higher for MRI (94%) but reasonably similar to the subjective method (88%). Agreement between subjective and objective TVS methodologies was fair to moderate. As regard the agreement between MRI and TVS, subjective measurement performed slightly better comparing to the objective method. A previous study found that a cut-off less than 20 mm from the outer cervical ostium to the lower margin of the tumor was correlated to the probability of cervical stromal invasion; however, subjective assessment seemed to perform significantly better (12).

The main drawback of our study is a small number of referred patients meeting the inclusion criteria (n=82), even though the recruitment has taken place in three centres with oncology care. The small sample size may jeopardize generalization of results and analysis of reproducibility. Additionally, the study may be underpowered to determine the accuracy of pre-surgical staging regarding cervical invasion, due to lack of patients with advanced diseased and unsuitable for surgery. The number of enrolled patients, which did not perform MRI (12/82, 15%) is not negligible and must also be taken into account. Another identified pitfall was the impossibility of performing an inter-observer agreement study of ultrasound and MRI measurement techniques. Regarding to the shortcomings of TVUS, the evaluation is examiner dependent and might be influenced by the equipment and patient profile. An additional drawback is the tumor profile itself: a large polypoid bulky endometrial cancer may lead to an overestimation on myometrial invasion due to the stretching effect on the surrounding myometrium, while a small uterus with a supposedly thin, hypoechoic or non-defined endometrial stripe may be deeply infiltrative (6).

Strengths of our study include the prospective multicentre design and the fact that both gynecology and radiology experts performing imaging staging were blinded to the remaining results. Although the sample size remains below the expected for extracting robust evidence for clinical applicability and decision-making, still it is comparable to the current scientific papers published regarding this issue. The authors believe that a large multicentre trial with more controlled variables (namely technique expertise and patient confounders) will be largely beneficial, since it will allow an increase in the sample size as well as a better representation of the Portuguese population. Although this was not a trial conducted to evaluate reproducibility, it was interesting to understand the differences in applying the same protocol in different hospital settings around the country and performed by professionals with different levels of expertise. Moreover, a classical reproducibility study, implying the performance of TVS by different experts could raise issues of compliance and enrollment due to discomfort of a long examination, possibly compromising the final sample size. Still, the authors believe it would be important to address this matter in further studies.

Ultrasonography is a commonly available, non-invasive and low-cost modality, standing as a reliable alternative to MRI, especially in medium and low income countries where MRI is not promptly available and costs are an important issue. Additionally, its use will potentially impact surgical planning, cost and time management in the operating room, obviating the need for frozen section exam and allowing an accurate selection of low and intermediate risk cases eligible for sentinel lymph node technique. According to recent international guidelines, sentinel lymph node biopsy is an acceptable alternative to systematic lymphadenectomy for lymph node staging even in stage I/II high-intermediate and high-risk disease (4).

In conclusion, pelvic ultrasound can play an important role in the preoperative staging of endometrial cancer, presenting a high sensitivity for the myometrial invasion assessment with a fair to moderate agreement with MRI and a better, although still moderate, agreement with MRI in the assessment of cervical invasion. However, further studies with larger sample sizes including several referral centers and assessing reproducibility are needed in order to reinforce our conclusions.

## Data availability statement

The raw data supporting the conclusions of this article will be made available without undue reservation. Further inquiries can be directed to the corresponding author.

## Author contributions

MR conceived and designed the analysis, collected the data and wrote the paper. CC-S conceived and designed the analysis, contributed to data and analysis tools and performed the analysis. JB and AC performed a critical revision of the article for important intellectual content and approved the version to be published. All authors contributed to the article and approved the submitted version.
